# Complete mitochondrial genome of the Cumberland slider (*Trachemys scripta troostii,* Testudienes: Emydidae) in Korea

**DOI:** 10.1080/23802359.2021.1902410

**Published:** 2021-03-19

**Authors:** Gayeon Ryu, Jae-I Moon, Yun-Ju Song, Jaehong Park, Seung-Min Park, Jae Hyeok Choi, Ha-Cheol Sung, Dong-Hyun Lee

**Affiliations:** a School of Biological Sciences and Biotechnology Graduate School, Chonnam National University, Gwangju, Korea; b Research Center of Ecomimetics, Chonnam National University, Gwangju, Korea; c Department of Biological Sciences, College of Natural Sciences, Chonnam National University, Gwangju, Korea

**Keywords:** *Trachemys scripta troostii*, mitochondrial genome, phylogenetic tree, phylogenetic analysis

## Abstract

The complete mitochondrial (mt) genome of *Trachemys scripta troostii* was sequenced and was characterized, which comprised 37 genes (13 protein-coding genes, 22 transfer RNAs, and 2 ribosomal RNAs) and a non-coding control region. Phylogenetic analysis based on the full mt genome indicated that *T. s. troostii* was more closely related to *T. scripta* from Canada than to *T. s. elegans* from China or *T. s. scripta* fom China. This is the first complete mt genome from *T. s. troostii*, which provides data for further study of phylogeny in Emydidae.

The Cumberland slider (*Trachemys scripta troostii*) is native in Cumberland and Tennessee Rivers, from south-eastern Virginia and Kentucky to north-eastern Alabama in the United States (Cagle 1950). *Trachemys scripta* was traded by pet market worldwide – including Austria, Poland, and Korea, but some were deliberately released to the field (Kitowski 2013; Kleewein 2014; Koo et al. 2020). We collected the turtle which is not native in Korea. With some morphological characters such as the shape of head, and the color pattern of carapace and plastron bone, we identified it as *T. s. troostii*. According to a recent study, about 1.2% of non-native turtles corresponded to *T. s. troostii* in Korea (Koo et al. 2020). Compared to other *T. scripta* subspecies, there is lack of genetic research about *T. s. troostii* in the world. There was a phylogenetic study about *T. s. troostii* by using three mitochondrial (mt) DNA fragments (12 s ribosomal RNA, Cytochrome b + 29 bp DNA coding for transfer RNA-Threonine, NADH-ubiquinone oxidoreductase chain 4 L/NADH-ubiquinone oxidoreductase chain 4) (Vamberger et al. 2020). However, its complete mt genome has not been reported yet. We first sequenced the complete mt genome of *T. s. troostii*, and this data can be used for further phylogenetic and evolutionary studies.

The *T. s. troostii* specimen was collected from Gwangju (35°10′28.1″ N; 126°54′35.9″ E), Korea. We extracted the total genomic DNA from the tail using the DNeasy Blood & Tissue kit (Qiagen, Valencia, CA) according to the manufacturer’s protocol and the extracted DNA sample was deposited at the Museum of Wildlife, located in Research Center of Ecomimeitcs, Chonnam National University, Korea (Specimen accession number: 2020-RCE-TST001; shcol2002@chonnam.ac.kr). We constructed the complete mt genome by primer walking method with the primer pairs (Supplementary Table 1). The complete mt genome was sequenced using Applied Biosystems 3730XL DNA Analyzer (Applied Biosystems, Foster City, CA, USA). We used Needleman-Wunsch algorithm on NCBI to align the sequences, checked quality control from chromatogram data provided by Bionics (Seoul, Korea) which offers DNA sequencing services. Each read was aligned and annotated by comparing *T. scripta* mt genome (Accession No. FJ392294), *T. s. elegans* mt genome (Accession No. KM216748), and *T. s. scripta* mt genome (Accession No. KM216749) in GenBank.

The complete mt genome of *T. s. troostii* was 16,810 bp in length deposited in GenBank (Accession No. MW122292), and contained 13 protein-coding genes (PCGs), 22 transfer RNA (tRNA) genes, 2 ribosomal RNA (rRNA) genes (12 s and 16 s), and a putative non-coding control region (NCR). Twelve PCGs, 14 tRNAs, 2 rRNAs were predicted to be located in the heavy strand, whereas 1 protein-coding gene (NADH dehydrogenase subunit 6) and 8 tRNAs were encoded in the light strand. The nucleotide composition of the *T. s. troostii* from Korea mt genome (A = 34.3%, C = 25.9%, G = 12.9%, and T = 27.0%) was same with that of *T. scripta* from Canada (A = 34.3%, C = 25.9%, G = 12.9%, and T = 27.0%), and similar with that of *T. s. elegans* from China (A = 34.2%, C = 25.9%, G = 12.9%, and T = 27.0%), and *T. s. scripta* from China (A = 34.2%, C = 25.9%, G = 12.9%, and T = 27.0%). The sequence comparison with *T. s. troostii* from Korea and *T. scripta* from Canada indicated a 99.97% sequence identity. But the sequence identity was 99.68% between *T. s. troostii* from Korea and *T. s. elegans* from China, and was 99.41% between *T. s. troostii* from Korea and *T. s. scripta* from China.

In order to investigate the phylogenetic position of *T. s. troostii* from Korea, the full mt genome sequences of 12 turtle species were extracted from Genbank. Based on other studies (Krenz et al. 2005; Russell and Beckenbach 2008), we chose *Pelomedusa subrufa* as an outgroup which belongs to the suborder ‘Pleurodira.’ The sequences were aligned using MUSCLE algorithm (Edgar 2004), and the phylogenetic tree was constructed using Maximum Likelihood (ML) method with Tamura-Nei model as the nucleotide substitution type in MEGA X software (Tamura and Nei 1993; Kumar et al. 2018). It shows that every *T. scripta* subspecies is clustered in a monophyletic group. *T. s. troostii* from Korea is more closely related to *T. scripta* from Canada than to *T. s. elegans* from China or *T. s. scripta* from China (Figure 1). These data provide important molecular information for further studies about evolutionary analysis and can be used as a useful genetic marker for identification and ecological studies on *T. s. troostii*.

**Figure 1.**
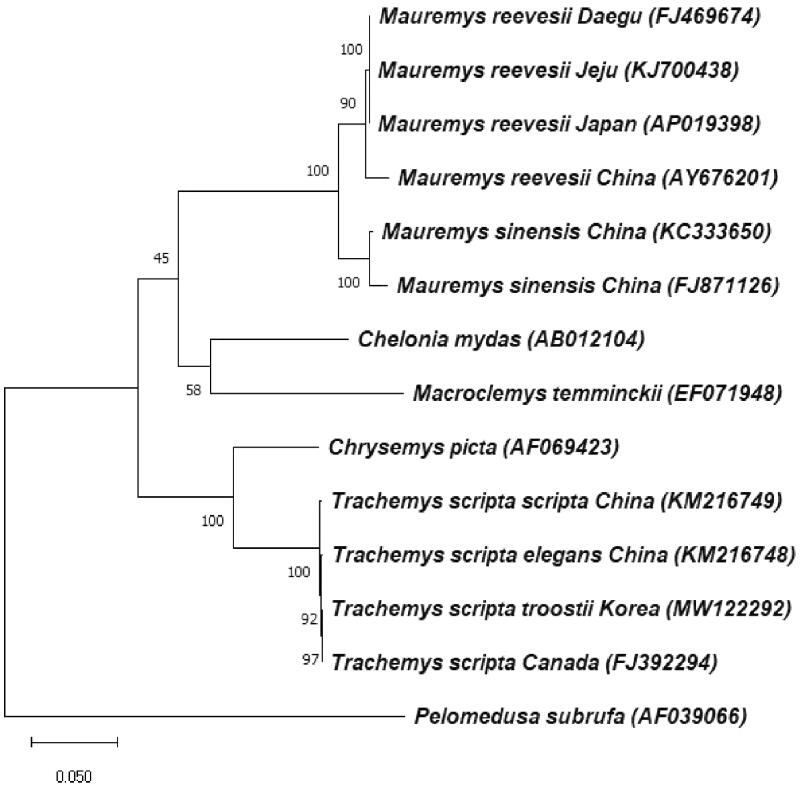
The maximum likelihood (1000 bootstrap replicates) tree based on complete mt genome data of *Trachemys scripta troostii* from Korea and other 13 species. *Pelomedusa subrufa* was set as outgroup. Ryu et al.

## Data Availability

GenBank accession number from the complete mitochondrial genome of *Trachemys scripta troostii* (MW122292) has been registered with the NCBI database (https://www.ncbi.nlm.nih.gov/).
